# Cultural Differences in the Relationship between Intrusions and Trauma Narratives Using the Trauma Film Paradigm

**DOI:** 10.1371/journal.pone.0106759

**Published:** 2014-09-09

**Authors:** Laura Jobson, Tim Dalgleish

**Affiliations:** 1 Medical Research Council Cognition and Brain Sciences Unit, Cambridge, United Kingdom; 2 University of East Anglia, Norwich, United Kingdom; 3 Cambridgeshire and Peterborough NHS Foundation Trust, Cambridge, United Kingdom; University College London, United Kingdom

## Abstract

Two studies explored the influence of culture on the relationship between British and East Asian adults’ autobiographical remembering of trauma film material and associated intrusions. Participants were shown aversive film clips to elicit intrusive images. Then participants provided a post-film narrative of the film content (only Study 1). In both studies, participants reported intrusive images for the film in an intrusion diary during the week after viewing. On returning the diary, participants provided a narrative of the film (delayed). The trauma film narratives were scored for memory-content variables. It was found that for British participants, higher levels of autonomous orientation (i.e. expressions of autonomy and self-determination) and self-focus in the delayed narratives were correlated significantly with fewer intrusions. For the East Asian group, lower levels of autonomous orientation and greater focus on others were correlated significantly with fewer intrusions. Additionally, Study 2 found that by removing the post-film narrative task there was a significant increase in the number of intrusions relative to Study 1, suggesting that the opportunity to develop a narrative resulted in fewer intrusions. These findings suggest that the greater the integration and contextualization of the trauma memory, and the more the trauma memory reflects culturally appropriate remembering, the fewer the intrusions.

## Introduction

Involuntary autobiographical memories are memories of personal events that consciously and spontaneously come to mind without any deliberate retrieval intention [1]. While they are common and frequently recalled in the daily life of healthy adults, in their more extreme form they have been found to be associated with a range of psychological disorders [2]–[5]. For instance, posttraumatic stress disorder (PTSD) is characterized by involuntary memory retrieval; the hallmark symptom of PTSD is the involuntary, intrusive recollection of memories of the traumatic experience [6]–[8]. Therefore, the psychological processes autobiographical remembering and information-processing have become central to the understanding of PTSD [4]. An account for these intrusive trauma memories has been offered by cognitive autobiographical memory models, such as the Self Memory System (SMS) [9], [10], and PTSD specific models, such as the cognitive model of PTSD [8] and the Dual Representation Theory (DRT) [7] (see [11] for a review).

The SMS [9], [10] posits that a motivational hierarchy of goals (the working self) regulates, encodes and integrates memories into an autobiographical knowledge base – a hierarchical database of memories with general summaries of lifetime periods at the top and increasingly specific details of individual events (event specific knowledge) at the bottom. This allows for autobiographical memories to have connections to lifetime periods and other general events. Voluntary retrieval of specific event details generally requires navigating down this hierarchy. Retrieval can also occur via ‘direct access’ to specific event representations in the memory hierarchy thus bypassing the hierarchical search that underpins voluntary retrieval. Memory integration into the autobiographical knowledge base allows for elaboration of the memory, which enhances the first retrieval route and inhibits the second. The SMS suggests that trauma can pose a threat to current goals to which the working self cannot adapt. Hence, there are no currently active goals that can be used to integrate and contextualize the trauma memory into the autobiographical knowledge base. Instead the trauma memory remains an event specific representation. Consequently it is difficult to retrieve the trauma memory using the first retrieval route as it lacks the connections to other autobiographical memories. Rather it is activated, involuntarily, via the second retrieval route.

Ehlers and Clark [8] similarly suggest that the PTSD trauma memory is not well integrated or contextualized. They suggest that for those with PTSD ‘conceptual’ processing (which places the trauma information in such a way that it is coherent, chronological, meaningful and has context) is impaired while ‘data-driven’ processing (which involves sensory information) dominates during a traumatic event. The DRT [7] suggests that there is the Situationally Accessible Memory (SAM) system and Verbally Accessible Memory (VAM) system (more recently the VAM system has been referred to as contextual memory [C-memory] and the SAM system has been referred to as low-level sensation-based memory [S-memory]. See [12] for further details). These two systems operate in parallel but one system can take precedence over the other at different times. The SAM system is limited to material that was encoded using lower level perceptual processing of the traumatic scene, such as sights and sounds. Thus, it can only be accessed involuntarily through situational reminders of the trauma. The VAM system includes material that was consciously processed during the traumatic event and can be accessed through voluntary recall and described verbally. Ideally, SAMs are integrated with VAMs to form a coherent contextualized account of the trauma event. However, under intense stress the processing that leads to VAMs is impaired resulting in the domination of the SAM system. As a result of very little information being encoded in the VAM system, memories of the trauma are repeatedly brought to mind as sensory and emotional fragments. As the SAM system does not use a verbal code, these trauma memories are difficult to communicate voluntarily to others. Furthermore, the memories tend not to interact with and, thus, get updated by other autobiographical knowledge [7].

All of the above models posit that PTSD intrusions are the result of a lack of memory integration and contextualization. Holmes and Bourne [4] suggest this may result from trauma events unfolding very rapidly reducing the time available for sufficient verbal, conceptual processing. Rather the individual focuses on the sensory, visuospatial information as it may assist in current and future survival. Manipulating the processing of trauma should therefore influence the development of intrusions. Encoded events are unlikely to intrude if there is sufficient balance and usage of verbal and visuospatial processing of trauma information. However, when there is an increase in the balance of visuospatial relative to verbal processing (or impairments in verbal processing) then it is likely that the individual will experience more intrusions. Alternatively, if there can be a processing shift to increase verbal processing (or reduce visuospatial processing) then this may protect against intrusions [4].

In order to examine these propositions the trauma film paradigm has been used as an experimental analogue of witnessing real trauma and of the subsequent intrusions suffered in PTSD [4]. This paradigm involves showing healthy participants short films depicting material that is considered traumatic according to the DSM-IV-TR (Criterion A1) [13]. Participants are typically given a diary following viewing of the film. In this diary participants monitor any subsequent symptoms consistent with a PTSD response (for example, intrusive memories of the film content; analogue flashbacks) (see [4]). Participants have been required in these empirical studies to engage in a concurrent task during the film which can be tailored to compete for perceptual (visuo-spatial) or verbal processing (see [6] for a review). Researchers have consistently found that when participants have engaged in tasks that required visuospatial processing (i.e. tasks that interfered with visuospatial encoding of the film) there was a reduction in subsequent intrusions of the film, relative to participants in a no-task condition [14]–[19]. Researchers that have investigated the influence of verbal processing on trauma film-related intrusions have found a less consistent pattern of results. Some researchers have found that, as expected, participants who engaged in a task that interfered with verbal, conceptual processing of the film material reported an increase in intrusive images, relative to participants in a no-task condition [3], [14], [16], [20]. However, other researchers have found a concurrent verbal task did not influence the frequency of intrusions [20] – [22] and in some cases even led to a decrease in the frequency of intrusions, relative to a no-task control condition [18], [23], [24]. Brewin [6] suggests that these inconsistent findings may be the result of the nature of the verbal task selected. For instance, some tasks may have been ineffective because they were insufficiently demanding. Nevertheless, there is evidence to support the notion that visuospatial processing and prohibition of conceptual integration of trauma information may be involved in the formation of intrusive trauma memories [6], [23].

While the trauma film paradigm has increased our understanding of the processes likely to be involved in PTSD, there is an important under-researched area in which this paradigm has potential value – investigating the influence of culture on the development and maintenance of PTSD. Western cultures value an independent and autonomous self. The goals associated with this aspect of self include being unique, expressing the self, realizing internal attributes, and promoting personal goals. In contrast, East Asian cultures tend to emphasize an interdependent self. The goals associated with this aspect of self include attending to and fitting in with others and the surrounding social context [25]. It is important to note that there is variation in the degree to which individuals exhibit an independent versus interdependent self-orientation both within and between Western and East Asian cultures (e.g., [26]). However, researchers have continuously demonstrated marked differences in self-construal between individuals from Western and East Asian cultures (e.g., [25]). Moreover, this cultural distinction has been found to have extensive effects on socialization and cognition [27], including information-processing (see [28]) as well as the content and organization of autobiographical memories. The Western perspective emphasizes sharing of self-relevant autobiographical memories and self-expression. In contrast, East Asian cultures discourage excessive self-focused remembering [27]. In support of this, autonomously oriented, self-focused, emotionally elaborate, lengthier accounts of personal events are more prevalent among Western cultures than East Asian cultures. East Asian cultures tend to downplay an autonomous orientation and rather focus on social interactions and significant others (e.g., [29]–[32], see also [33] for review). These cultural differences have been found to reflect more overarching differences in general narrative reporting rather than being unique to personal narratives. For instance, Wang and Ross [34] created a memory in the laboratory by showing American and Asian undergraduate students a fictional story and then asked them to recall the story. Systematic cultural differences were evident in the memory-content of the recollection of the story. Similarly, Han et al. [27] found that when Korean, Chinese and American children were asked to recall a narrated story, while objective story memory performance was equally accurate across cultures, memory-content culturally differed.

These cultural differences have been accounted for using the Self-Memory System (SMS) framework. The SMS suggests that, alongside the working self, the conceptual self regulates autobiographical remembering. The conceptual self is constitutive of an individual’s culture and contains socially-constructed schema and categories that define the self, other people and the relations between them [9]. Depending on cultural influences, the conceptual self emphasizes either independence or interdependence. Such differences influence the goals of the working self so that memories are encoded and integrated into the autobiographical knowledge base in ways that emphasize this cultural dominance. The integration of memories allows for the connection and elaboration of memories to other memories which also emphasize either independence or interdependence. Hence, when memories are retrieved, they are retrieved in such a way that cultural differences are evident (see [35], [36]). Therefore, memories that are well-contextualized and integrated into the autobiographical knowledge base provide greater opportunities for cultural influences to be exerted on the memory. Thus, when the memory is retrieved, cultural differences in memory-content variables are evident. For those from Western cultures, a well-integrated and contextualized memory is autonomously orientated and self-focused. In contrast, for those from East Asian cultures, a well-integrated and contextualized memory focuses on social interactions and others [31].

The aim of the two studies reported below was to investigate the influence of culture on the relationship between autobiographical remembering of the trauma film material and film-related intrusions. As outlined above, events that are encoded using verbal, conceptual processing and are well integrated and contextualized in autobiographical memory should be associated with fewer intrusions. Also, as outlined above, cultural differences in the conceptual self act as a constructive filter. This influences the manner in which information is encoded and represented in memory. It also serves as a reconstructive filter that shapes memory during retention and at the time of retrieval [34]. Thus, greater conceptual processing, integration and contextualization of a memory allows for cultural differences in self-construal to exert a greater influence on that memory [36]. Memories that are not contextualized and integrated provide less opportunity for the usual cultural differences (i.e. memory-content that emphasizes either independence or interdependence) to emerge. Given the findings of Han et al. [27] and Wang and Ross [34], such cultural influences should extend to the remembering of the content shown in the trauma film paradigm. Therefore, in the current two studies these memory-content variables (i.e. autonomous orientation, other vs. self focus and mention of social interactions) were used as evidence of the memory of the trauma film being integrated and contextualized. It was hypothesized that integration and contextualization of the memories of the film content would be associated with participants reporting fewer trauma film-related intrusions. Specifically, for those from Western cultures, greater emphasis on autonomy and more mention of the self in trauma film narratives would be associated with the reporting of fewer trauma film-related intrusions. In contrast, for those from East Asian cultures, downplaying of autonomous orientation and greater mention of others and social interactions in trauma film narratives would be associated with the reporting of fewer trauma film-related intrusions.

## Study 1

Study 1 investigated whether there was an association between the culturally expected memory-content qualities of the trauma film narratives and the reporting of trauma film-related intrusions. British and East Asian participants watched the trauma film and then provided an immediate narrative account of the film. Participants completed the intrusion diary in the week following watching the film [4]. Then when participants returned their diary the following week, they again provided a written account (delayed account) about their memory of the film. The memory-content variables (mention of others in relation to oneself, autonomous orientation and social interactions) of the two trauma film accounts were coded, as in previous cross-cultural research, to assess integration and contextualization of the memory (e.g., [31], [36]). Culturally appropriate integration and contextualization of the memory was indexed by the expected memory-content variables being evident in the narratives. It was hypothesized that integration and contextualization of the memory in the culturally appropriate way would be associated with the reporting of fewer intrusions. Specifically, it was predicted that for British participants, the trauma film narratives that contained greater emphasis on autonomy and self-focus would be associated with the reporting of fewer trauma film-related intrusions. In contrast, it was predicted that for the East Asian group, reduced autonomous orientation and greater mention of others and social interactions would be associated with the reporting of fewer trauma film-related intrusions. Second, cultural differences in self-construal act as a constructive filter influencing the manner in which information is initially encoded and represented in memory. Cultural differences in self-construal also serve as a reconstructive filter that shapes memory over the course of retention and at the time of retrieval [34]. Therefore, it was hypothesized that both the immediate and delayed trauma film narratives would culturally differ in terms of levels of autonomous orientation, self-focus and mention of social interactions. British participants would have significantly greater levels of autonomous orientation and self-focus and significantly less mention of social interactions than East Asian participants.

## Method

### Ethics Statement

Ethical approval for both studies was obtained from the Faculty of Medicine and Health Sciences University of East Anglia Ethics Committee. The safeguards that have been developed for use of the trauma film paradigm [4] were followed in the present studies. These included: non-inclusion of participants with past or present mental health difficulties; clear information to participants about film content prior to their participation; use of precautionary measures to deal with distressed participants (studies were conducted by clinical psychologists); clear information to participants about their right to withdraw from the study at any point; and provision of contact details to participants in the event that they have any concerns even after the study had finished.

### Participants

All participants were students at the University of East Anglia and were recruited via the Psychology Panel. Participants were 23 (18 females; 18 undergraduate, 5 postgraduate) white British students (i.e. participants were all born in Britain, spent the majority of their lives in Britain and identified their ethnicity as ‘white British’) and 22 (18 females; 17 undergraduate, 5 postgraduate) East Asian International students (Chinese *n* = 14, East Asian *n* = 8) (i.e. participants were all born in China or another East Asian country, had recently come to Britain as International students and identified their ethnicity as ‘East Asian’). Participants were informed of the content of the films and exclusion criteria included self-reported current or history of panic attacks, panic disorder, PTSD, major depressive episode, social phobia, psychotic episode, blood phobia and history of fainting. One participant was excluded based on these criteria (history of blood phobia). Participants were also excluded if they felt their English standard would hinder their ability to complete the tasks in English. No participants were excluded based on this criterion. Participants were paid £10 for their participation in the study.

### Trauma Film

A 10-minute trauma film based on Holmes, James, Coode-Bate, and Deeprose [37] was used. The Holmes et al. film comprised seven extracts of film footage of traumatic content, including graphic real scenes of human surgery, fatal road traffic accidents and drowning. In addition to the films used in Holmes et al. three clips that depicted Asian individuals involved in traumatic, distressing events were added to ensure all clips did not just include Western individuals. Four scenes depicted car accidents, two scenes depicted surgery and four additional scenes included drowning, genocide, an electricity pylon accident and a firework explosion. The trauma film was displayed on a 15 inch color monitor in a dark room and viewing distance was approximately 50 cm.

### Baseline Measures

#### Self-relevance for trauma depicted in the trauma film scenarios

To ensure British and East Asian participants were comparable in terms of personal exposure to the trauma experiences depicted in the film, single item self-report Visual Analogue Scales (VAS) ranging from 0 (*not at* all) to 10 (*extremely relevant*) were used to assess for personal exposure to the trauma events depicted in the scenarios (e.g. car accidents, surgery, drowning, accidents and war) [14].

#### Traumatic experience questionnaire (TEQ)

A 12-item checklist adapted from the criterion A list of the Posttraumatic Diagnostic Scale (PDS) [38] was also used to assess past experience of personal trauma. Participants indicated whether they had experienced or witnessed each of the traumatic events listed (i.e. accident, natural disaster, military combat and war, child sexual abuse, imprisonment, torture, life threatening illness and sexual and non-sexual assault). Events selected were summed and scores ranged from 0 (*no traumatic events experienced or witnessed*) to 12 (*each and every type of traumatic event experienced or witnessed*).

#### Depression Measure

Depression was assessed using Part-II of the Hopkins Symptom Checklist-25 (HSCL-25) [39] which contained 15 items that measured symptoms of depression. The measure required individuals to indicate how much each symptom has bothered or distressed them during the past week using a 4-point Likert scale ranging from 1 (*not at all*) to 4 (*extremely*). The HSCL-25 has been consistently shown to correlate with major depression across a range of different populations and has been used extensively in cross-cultural studies (e.g. [40]). The HSCL-25 has been shown to possess high internal consistency, with high test-retest reliability and adequate inter-rater reliability [39].

#### Personal narrative

To ascertain that the cultural differences found in previous research were present in the personal narratives of the East Asian and British participants selected (as in Han et al. [27]), participants were asked to write in detail, including thoughts, feelings and reflections, about two memories of events from any period of their lives that were personally important to them, both at the time of occurrence, and in retrospect [30]. Memories were coded for a) memory focus, b) other vs. self focus, c) autonomous orientation, and d) social interactions, as outlined below.

### State Measures

Before and after the film, mood (fearful, horrified, feelings of helplessness, depressed and angry) was rated by participants using VAS from 0 (*not at all*) to 10 (*extremely*) for feelings “right now” both pre- and post-film [14]. A composite mood score was computed, as in Bourne et al. [14], by calculating the mean score across each of the five emotions both pre- and post-film. After watching the film, participants also rated their distress and how much attention they had paid to the film on similar scales.

### Memory Measures

#### Involuntary intrusion diary

As in previous studies (e.g., [14], [41], [42]), participants were given a diary to record any image-based intrusions of film content during the seven days following the film session. Participants were instructed (verbally and via written instructions in the diary) that intrusions were “any memory of the film (or part of the film) that appear apparently spontaneously in your mind. Do not include any memories of the film that you deliberately or consciously bring to mind”. Participants were asked to record each day (which was divided into morning, afternoon and evening) all intrusions immediately after they occurred (whenever possible) and to set aside a regular time each day to check whether their diary was up-to-date as a way of ensuring intrusions were not omitted if it had been impractical to write down an intrusion immediately. If participants experienced no intrusions they were also required to record this. Participants were also instructed to describe the content of the intrusion so as to ensure that the intrusion was related to the film [14]. The approach employed by Bourne et al. [14] was used to assess the degree to which participants remembered to complete the diary. A single item VAS was used to measure the extent to which participants forgot or omitted to record intrusions. The omissions scale ranged from 0 (*not at all*) to 10 (*completely true of me*).

#### Immediate narrative

Immediately following the film, participants were asked to write about the trauma film scenes in as much detail as they could; “Now please think about the film you just watched. You were asked to watch the film as if you were there, a bystander at the scene of the events, and to pay attention to the film as later there will be questions about film content. Please write in detail as much as you can about the film as if you had been at these events. As you write do not worry about punctuation or grammar, just write as much as you can and include thoughts, feelings, reflections, etc.” [43].

#### Delayed narrative

When participants returned the following week, they were again asked to write about the trauma film scenes in as much detail as they could. Participants were instructed to “Now please think about the film you watched last week You were asked to watch the film as if you were there, a bystander at the scene of the events, and to pay attention to the film as later there will be questions about film content. Please write in detail as much as you can about the film as if you had been at these events. As you write do not worry about punctuation or grammar, just write as much as you can and include thoughts, feelings, reflections, etc.”

#### Recognition and recall

Two memory tests were used, as in Bourne et al. [14], to evaluate voluntary memory of the film. A 16-item ‘yes/no’ forced choice recognition test (e.g. Scene 1: A little girl has blood coming out of her ear after being hit by a car, Scene 5: The phone smashes as it hits the ground) and a 14-item cued recall test (e.g. What part of the face was being operated on in Scene 2?, What explodes in the face of the children in Scene 8?) for specific details were used.

### Demographics

All participants completed a demographic information sheet, which gathered information about age, gender, ethnicity, level of education and English, length of time they had lived in the UK and a rating scale assessing how difficult participants had found the study. The ‘I am’ [44] was included to examine cultural differences in the individual’s sense of self [45]. Participants were asked to provide 10 statements in response to the question “Who Am I?” Responses were coded as either an independent (private) self-statement or interdependent self-statement (collective or public) [46], [47]. Statements were coded as independent if the responses referred to personal qualities, attitudes, beliefs, or behaviors that were not related to other people (e.g., “kind”). Statements were coded as interdependent if they were collective self-statements (e.g., “Chinese”) or statements that pertained to interdependence, friendship, relationships or to the sensitivity of others (e.g., “in love”). An ‘independent ratio’ was calculated for each participant. This was calculated by dividing the total number of statements provided by the total number of independent statements provided. The initial coding was conducted by the first author. Inter-rater reliability was established by twenty percent of the statements being coded by an independent rater from an East Asian cultural background. Inter-rater reliability was good (*r* = .81). Discrepancies between raters were resolved through discussion.

### Coding of Memory-Content Variables

The memory-content variables of the two life event narratives and of the immediate and delayed trauma film narratives were analyzed based on a coding scheme that has been used extensively in previous studies [27], [29], [31], [45], [48], [49]. All memories were initially coded by the first author. As in this previous research, twenty percent of each data set was also coded by the independent rater for inter-rater reliability estimates. Again discrepancies between raters were resolved through discussion. The inter-rater reliabilities (*r*) between the independent rater and the initial rater ranged from.72 to.90.

#### Memory volume

The total number of words used by each participant was counted for each memory to index memory volume. As the memory variables, autonomous orientation, other-self focus and social interactions (outlined below), were calculated based on frequencies in coding; length of the narratives may have influenced the coding of these variables [31]. To address this, each participant’s variable total was divided by the length of their narrative.

#### Memory focus

Personal memories were coded into personal or social themes. Personal experiences were associated with objects or events not particularly related to other people, for example, academic/employment achievement or sporting endeavors. Social events were about collective activities of the family, workplace, community or other social groups.

#### Autonomous orientation

Autonomous orientation is a distinct memory variable in that it assesses the role of internal state and thus, suggests motives for one’s remembering and one’s personal meaning given to an event [27]. Any tendency of participants’ to express self-determination and autonomy in their memories was indexed using the autonomous orientation variable – a narrative content analysis scheme developed by Wang and Leichtman [48]. The number of occurrences of the following instances was counted and combined to produce an autonomous orientation score for each participant; (a) personal needs, desires or preferences; (b) personal dislikes or avoidance; (c) personal evaluations, judgments or opinions regarding other people, objects or events; (d) retaining control over one’s own actions and resisting group or social pressure; and (e) personal achievement or competency. Each participant’s total autonomous orientation score was divided by the number of words used in their narrative.

#### Other-self ratio

The other-self ratio has been used in previous research as an index of the degree to which participants provide non-egotistic memories and thus, their social orientation [31]. Other-self ratio involved counting the number of times participants mentioned other people and themselves in their memories. An ‘other-self ratio’ was calculated for each participant by dividing total other by total self. Each participant’s total ‘other-self ratio’ was then divided by the number of words used in their narrative.

#### Social interactions

The number of references that involved social interactions or group activities were counted and totaled for each participant. Each participant’s total number of social interactions was then divided by the number of words used in their narrative.

### Procedure

Participants first provided informed consent to participate in the research. All participants were tested individually. The experimenter was not present in the room during the film or whilst the participant completed all questionnaires and tasks. The experimenter sat in an adjacent room to provide further instruction/clarification if required. Participants completed the baseline assessments, pre-film mood rating and provided two personally important autobiographical memories. Following this, all participants watched the trauma film. Participants were instructed to pay close attention during the film and to imagine they were a bystander present and involved at each of the scenes. They were asked not to look away or close their eyes and to pay close attention because there would be questions following the film. Immediately, after the film participants provided an account of the film and completed ratings of post-film mood and of attention paid to the film. They were then shown how to complete the intrusion diary. After seven-days participants returned the diary and took part in the follow-up session. The follow-up session included writing about their memory of the film and completing the film-related recognition and recall tasks. Participants also completed the ‘I am’ task and provided demographics.

## Results

### Participant Characteristics

Participant characteristics are presented in Table 1. The groups did not differ significantly in terms of age, gender, self-reported task difficulty, or self-reported remembering to complete the diary. The East Asian group, unsurprisingly, had been in the UK significantly less time than the British group and reported significantly lower levels of English language ability than the British group. Given the potential influence these group differences may have had on subsequent findings, all analyses were also conducted including self-rated English skill ability and length of time in the UK as covariates. In each instance, a similar pattern of results emerged to that reported below. As expected, the British group had a significantly higher independent sense of self ratio on the ‘I am’ than the East Asian group. The groups were comparable in terms of depression scores and did not differ significantly in their previous exposure to trauma, or in the self-relevance of the trauma types presented in the film (see Table 1 for all *t* test statistics).

**Table 1:** Participant Characteristics and Group Means for Remembering of the Trauma Film Material for Study 1.

	British	East Asian	*t*(41)
Demographics			
Age – years	23.74 (5.93)	20.97 (5.89)	1.19
Time in UK – years	16.57 (11.52)	1.67 (1.38)	5.88**
Self-reported English ability	8.78 (1.13)	7.35 (1.53)	3.57**
Self-reported task difficulty	3.83 (2.49)	3.30 (1.62)	.83
‘I am’ independence ratio	.69 (.24)	.51 (.31)	2.12*
Forgot to complete diary	2.09 (2.11)	3.38 (4.72)	1.19
Baseline Measures			
Depression	23.70 (5.47)	25.19 (6.51)	.86
Life trauma exposure	1.26 (1.42)	1.36 (1.14)	.27
Car accident exposure	3.22 (3.04)	2.86 (2.29)	.44
Surgery exposure	4.35 (3.61)	3.18 (3.00)	1.18
Accident exposure	1.65 (2.53)	1.59 (2.20)	.09
Drowning exposure	3.22 (2.94)	2.73 (2.71)	.58
War exposure	1.78 (2.33)	1.55 (1.95)	.37
Personal Narratives			
Total volume	118.00 (40.30)	106.76 (47.07)	.86
Personal focus	1.21 (.78)	.71 (.72)	4.85^a^ *
Autonomous orientation	.14 (.06)	.09 (.05)	10.12^a^**
Other-self ratio	.02 (.02)	.04 (.04)	4.82^a^ *
Social interactions	.04 (.02)	.08 (.04)	11.47^a^**
State Measures			
Pre-film mood	.43 (.50)	.60 (.82)	–
Post-film mood	1.37 (.84)	2.04 (1.45)	–
Post-film distress	2.35 (1.53)	3.30 (2.25)	1.67
Attention	9.13 (.92)	8.85 (.77)	1.11
Remembering of Trauma Film Material			
Intrusions	4.96 (3.14)	3.23 (2.84)	1.90
Recall	10.96 (1.80)	10.10 (1.95)	1.52
Recognition	10.96 (1.40)	10.55 (1.50)	.93
Trauma Film Narrative – Immediate			
Volume	129.83 (63.06)	110.38 (42.70)	–
Autonomous Orientation	.07 (.03)	.06 (.03)	–
Other-self ratio	.01 (.02)	.01 (.01)	–
Social Interactions	.004 (.01)	.01 (.01)	–
Trauma Film Narrative – Delayed			
Volume	97.61 (30.37)	86.90 (38.69)	–
Autonomous Orientation	.04 (.03)	.04 (.03)	–
Other-self ratio	.02 (.02)	.03 (.04)	–
Social Interactions	.003 (.01)	.01 (.01)	–

a Results from the follow-up multiple univariate ANOVA analyses [*F*(1,43)].

*p<.05 **p<.01.

### Personal Narratives

Scores for each of the memory-content variables were summed across the two personal memories. As seen in Table 1, the groups did not differ significantly in terms of memory volume. A multivariate analysis (MANOVA) was then used to compare East Asian and British participants with memory-content variables (personal focus, autonomous orientation, other-self ratio and social interactions) as the dependent variables. The multivariate effect of Group was significant, Λ = .73, *F*(4, 40) = 3.70, *p* = .01, η_p_^2^ = .27. Given the memory-content variables were proposed to represent an underlying construct (i.e. self-construal), the MANOVA was followed up with discriminant analysis [50]. This revealed one discriminant factor, canonical *R* ^2^ = .27, which significantly differentiated the cultural groups, χ^2^(4) = 12.90, *p = *.01. The correlations between outcomes and the discriminant function revealed that autonomous orientation (*r* = .80) and social interactions (*r* = −.85) loaded highly onto the function and personal theme (*r* = .55) and other-self ratio (*r* = −.55) loaded moderately onto the function. Autonomous orientation and personal theme are independent markers. Therefore, both these variables had a similar positive direction of relationship with the factor. In contrast, other-self ratio and social interactions are both interdependent markers. Therefore, they both had a similar negative direction of relationship with the factor. Hence, the expected cultural differences in personal autobiographical remembering were evident. Follow-up multiple univariate ANOVAs were also conducted and the results of these analyses are presented in Table 1.

### State Measures

A mixed 2 (group; East Asian vs. British) x 2 (time, pre-film vs. post-film) analysis of variance (ANOVA) found a significant time main effect; participants scored significantly higher on the mood measures following the film, *F*(1, 43) = 45.36, *p*<.001, η_p_^2^ = .51. The group main effect, *F*(1, 43) = 3.74, *p = *.06, η_p_^2^ = .08, and interaction, *F*(1, 43) = 2.01, *p = *.16, η_p_^2^ = .05, were not significant. The groups did not differ in terms of post-film distress or attention paid to the film (see Table 1 for *t* test statistics).

### Intrusions of Film-Related Material

As shown in Table 1, East Asian and British participants did not differ significantly regarding the number of film-related intrusions during the week following viewing the film as self-recorded in the diary. The groups also did not differ significantly in terms of recognition and recall suggesting that objective memory performance was equally accurate across cultures (see Table 1).

### Trauma Film Narratives

In terms of length of the trauma film narratives, while the cultural groups did not differ significantly, *F*(1, 43) = 2.31, *p* = .13, η_p_^2^ = .05, the immediate narratives were significantly longer than the delayed narratives, *F*(1, 43) = 8.03, *p*<.01, η_p_^2^ = .16. The interaction between time and group was not significant, *F*(1, 43) = .87, *p* = .36, η_p_^2^ = .02. A 2 (time: immediate vs. delayed) x 2 (group: East Asian vs. British) x 3 (memory-content variables: autonomous orientation, other-self ratio, social interactions) mixed ANOVA, with proportion of memory-content variable as the dependent variable was conducted. Unexpectedly, there was no significant group main effect, *F*(1, 43) = .02, *p* = .91, η_p_^2^<.001. Additionally, the variable x group interaction, *F*(2, 86) = .25, *p* = .78, η_p_^2^<.01, time x group interaction, *F*(1, 43) = .20, *p* = .66, η_p_^2^<.01, and three-way interaction, *F*(2, 86) = .58, *p* = .56, η_p_^2^ = .01, were all non-significant. The time x variable interaction was significant, *F*(2, 86) = 22.29, *p*<.001, η_p_^2^ = .34. The immediate narratives had significantly greater proportion of autonomous orientation, *t*(44) = 4.70, *p*<.001, *d* = 1.00, and significantly lower proportion of other-self ratio, *t*(44) = 3.90, *p*<.001, *d* = 0.63, than the delayed narratives. Mention of social interactions did not significantly differ between the immediate and delayed narratives, *t*(44) = .55, *p* = .59, *d* = 0.10.

### Correlations between Trauma Film Narrative Properties and Trauma Film-Related Intrusions

Pearson’s correlation coefficients were used to investigate the relationships between film-related intrusions and trauma film narrative memory-content variables for both immediate and delayed trauma film accounts. To control for Type I error associated with multiple correlations Bonferroni correction was applied (α = .017) and given the hypotheses, one-tailed tests were selected. In Table 2 it is shown that for both cultural groups, frequency of film-related intrusions was not correlated significantly with any of the memory-content variables of the immediate trauma film narrative. Regarding the delayed narrative, for the British group a higher frequency of film-related intrusions was correlated significantly with lower levels of autonomous orientation and higher levels of other-self ratio. For the East Asian group, a higher frequency of intrusions was correlated significantly with higher levels of autonomous orientation and lower levels of other-self-ratio. These correlation coefficients differed significantly.

**Table 2:** Summary of Correlation Coefficients between Memory-Content Variables and Number of Film-Related Intrusions (and Z score comparisons of the correlation coefficients) for each Group for Study 1.

	British Intrusions	East Asian Intrusions	Z score
Trauma Narrative - Immediate			
Autonomous orientation	−.08	−.23	0.48
Other-self	−.09	−.30	0.68
Social interactions	−.10	.02	0.38
Trauma Narrative - Delayed			
Autonomous orientation	−.43*	.45*	2.95*
Other-self	.46*	−.51**	3.31**
Social interactions	−.12	−.13	.03

*p<.05 **p<.01.

## Discussion

This study investigated whether integration and contextualization of memories of a trauma film were associated with trauma film-related intrusions. Integration and contextualization was indexed by culturally expected levels of autonomous orientation, social interactions and egocentricity being present in the narratives. It was first hypothesized that culturally appropriate integration and contextualization of the memory would be associated with fewer intrusions. In support of this, British participants who had lower levels of autonomous orientation and self-focus in their delayed narratives reported a higher frequency of film-related intrusions. In Western autobiographical remembering autonomous orientation and self-focus are valued [31]. In contrast, among East Asian participants, those with higher levels of autonomous orientation and less mention of others reported a higher frequency of film-related intrusions. In East Asian cultures autonomous orientation is downplayed and other-focus is emphasized [31]. Interestingly, there was no significant relationship between the memory-content variables associated with the immediate trauma film narrative and film-related intrusions. Thus contextualization and integration of the memory may take time and such differences may not emerge immediately following encoding. Rather rehearsal may be required to contextualize and integrate the memory and to allow for differences in self-construal to serve as a reconstructive filter that shapes memory over this period of retention [34].

Second, it was hypothesized that the immediate and delayed trauma film narratives would culturally differ in levels of the memory-content variables measured. While the British and East Asian International students differed in their autobiographical remembering of personal events, these cultural differences were not evident in the immediate or delayed trauma film narratives. Therefore, there was no support for the second hypothesis. It is uncertain why this was the case as previous researchers have found systematic cultural differences in the remembering of non-self-relevant fictional material. It is possible that the task (i.e. trauma scenes vs. a fictional story) influenced findings. Wang and Ross [34] employed a fictional story book called “Bear Goes to the Market”. This book contained illustrations in an explicit attempt to encourage personal interpretations of events in order to allow cultural effects to emerge. The storyline included both social scenarios and cognitive and affective responses in an attempt to derive cultural differences in encoding and recall. In contrast, the trauma film contained distressing emotional content and the storylines were not explicitly designed to encourage cultural differences in remembering to emerge. Timing of the recall test may have influenced findings. Han et al. [27] showed “Bear Goes to the Market” to participants on Day 1 and then tested recall on Day 2. Therefore, in the current study cultural differences may not have been found in the immediate narrative because a period of time was required for cultural differences to emerge [27]. In support of this, the correlations between the memory-content variables and frequency of intrusions were only found for the delayed narrative and not the immediate narrative. The question emerges however, why cultural differences were not evident in the delayed narrative. It is possible that the immediate narrative in some way disrupted processing. For instance, participants may have provided a delayed narrative that was based on the memory of their immediate narrative rather than on their memory of the film. Further research is required to investigate these possibilities.

This appears to be the first study to investigate trauma film intrusions in non-Western samples. East Asian and British participants did not differ significantly regarding the number of intrusions. This suggests that the trauma film is a useful paradigm to use in other cultural groups and to examine cultural differences in the processing of trauma. This study suggests that greater integration and contextualization of the trauma memory may be associated with fewer film-related intrusions experienced by participants. British participants who had lower levels of autonomous orientation and self-focus in their narratives of the trauma film reported a higher frequency of film-related intrusions. In contrast, East Asian participants with higher levels of autonomous orientation and a reduced mention of others in their narratives of the trauma film reported a higher frequency of film-related intrusions.

## Study 2

Direct efforts to enhance conceptual post-memory integration have been found to reduce the frequency of trauma film-related intrusions [51]. Krans et al. [51] conducted a study that aimed to enhance memory integration by administering a verbal recognition memory test for one part of the film directly after viewing in order to allow trauma film material to be rehearsed in a structured way. They hypothesized that such a task should enhance the formation of a memory that is verbally accessible, contextualized, organized, and able to be deliberately retrieved and thus, associated with fewer trauma film-related intrusions. Their findings supported this hypothesis. Additionally, participants’ performance on a cued-recall memory test administered during the one-week follow-up session was improved. They concluded that completing this memory recognition task immediately post-viewing resulted in the film material being better contextualized and integrated in autobiographical memory. Therefore, the immediate narrative provided by participants in Study 1 may have similarly enhanced conceptual post-memory integration of the trauma film material. That is, developing a narrative about the film content immediately after viewing may have served a similar function to Krans et al.’s verbal recognition memory test. Therefore, the first aim of Study 2 was to investigate the effect of removing the immediate narrative on the frequency of intrusions during the week. It was predicted that by removing the initial narrative there would be an increase in the number of trauma film-related intrusions and reduced performance on the recognition and free recall memory tasks (relative to Study 1). The second aim of Study 2 was to investigate whether the relationships between memory-content qualities and frequency of intrusions found in Study 1 could be replicated. Third, cultural differences in self-construal are proposed to act as a reconstructive filter that influences memory over the period of retention and at the time of retrieval [34]. However, Study 1 provided no evidence to suggest cultural differences in the memory-content qualities of the trauma film narratives. Therefore, the final aim of this study was to again investigate whether the delayed trauma film narrative would culturally differ in terms of levels of autonomous orientation, self-focus and mention of social interactions.

## Method

### Participants

As in Study 1, all participants were students at the University of East Anglia and were recruited via the Psychology Panel. Twenty-one (13 females; 14 undergraduate, 7 postgraduate) white British participants and 32 (21 females; 16 undergraduate, 16 postgraduate) East Asian International student participants (Chinese *n* = 17, East Asian *n* = 12, Japanese *n = *3). As in Study 1, exclusion criteria included self-reported current or history of panic attacks, panic disorder, PTSD, major depressive episode, social phobia, psychotic episode, blood phobia and history of fainting. No participants were excluded based on these criteria. Participants were also again excluded if they felt their English standard would hinder their ability to complete the tasks in English. One participant was excluded based on this criterion.

### Procedure

The design, measures and procedure were identical to that used in Study 1. However, participants were not required to provide an account of the trauma film immediately post-viewing the film. The only account of the trauma film provided was at the one-week follow-up session.

### Coding

Memories were coded by the first author as in Study 1. An independent rater, who was East Asian, coded twenty percent of each data set for inter-rater reliability estimates. Discrepancies between raters were resolved through discussion. Inter-rater reliability for the independent self ratio on the “I am” was good (*r* = .79) and the inter-rater reliabilities for the memories ranged between *r* = .70–1.00.

## Results

### Participant Characteristics

Participant characteristics are presented in Table 3. The groups did not differ in terms of age, gender, self-reported task difficulty, or self-reported remembering to complete the diary. The East Asian group had been in the UK significantly less time than the British group and reported significantly lower levels of English speaking ability than the British group. As in Study 1, all analyses were also conducted including self-rated English skill ability and length of time in the UK as covariates. A similar pattern of results to that outlined below emerged in each instance. As in Study 1, the British group had a significantly higher independent sense of self ratio on the ‘I am’ than the East Asian group. As shown in Table 3, the groups were comparable in terms of depression scores and did not differ in the self-relevance of the trauma types depicted in the trauma film, or in their previous exposure to trauma.

**Table 3:** Participant Characteristics and Group Means for Remembering of the Trauma Film Material for Study 2.

	British	East Asian	*t*(51)
Demographics			
Age – years	27.33 (10.55)	24.09 (5.37)	1.48
Time in UK – years	26.30 (9.39)	1.60 (1.79)	14.54**
Self-reported English ability	8.67 (1.31)	6.94 (1.32)	4.67**
Self-reported task difficulty	3.76 (2.23)	3.38 (1.58)	.74
‘I am’ independence ratio	.76 (.22)	.51 (.28)	3.34**
Forgot to complete diary	1.57 (1.40)	2.34 (1.52)	1.87
Baseline Measures			
Depression	24.10 (7.17)	26.33 (7.84)	1.04
Life trauma exposure	1.45 (1.10)	1.42 (1.23)	.08
Car accident exposure	3.70 (2.85)	3.58 (2.46)	.17
Surgery exposure	4.65 (3.34)	3.55 (2.98)	1.25
Drowning exposure	1.50 (1.96)	2.70 (2.36)	1.90
Accident exposure	3.75 (2.67)	4.36 (2.60)	.83
War exposure	1.35 (1.95)	1.97 (2.69)	.90
Personal Narratives			
Total Volume	117.14 (40.99)	111.44 (51.62)	.43
Personal Focus	1.10 (.83)	.78 (.71)	2.18^a^
Autonomous orientation	.13 (.05)	.08 (.05)	14.15^a^ **
Other-self ratio	.02 (.04)	.02 (.02)	0.14^a^
Social Interactions	.04 (.03)	.08 (.05)	13.05^a^ **
State Measures			
Pre-film mood	.91 (1.16)	1.21 (1.41)	.81
Post-film mood	2.03 (1.42)	3.59 (2.05)	3.06**
Post-film distress	3.24 (2.30)	3.91 (3.03)	.86
Attention	9.43 (.75)	8.94 (.91)	2.05
Remembering of Trauma Film Material			
Intrusions	8.38 (7.79)	9.28 (7.15)	.43
Recall	9.90 (2.45)	8.62 (2.22)	1.96
Recognition	9.90 (2.07)	9.48 (1.52)	.84
Trauma Film Memory-Content Variables			
Volume	146.43 (52.08)	107.25 (46.51)	2.86**
Autonomous orientation	.08 (.05)	.06 (.03)	5.62^a^ *
Social interactions	.005 (.01)	.02 (.02)	6.63^a^ *
Other-self ratio	.01 (.02)	.02 (.03)	1.59^a^

a Results from the follow-up multiple univariate ANOVA analyses [*F*(1,51)].

*p<.05 **p<.01.

### Personal Narratives

In Table 3 it is shown that the two groups did not differ significantly in terms of volume. As in Study 1, a MANOVA was used to compare East Asian and British participants with memory-content variables as the dependent variables. The multivariate effect of group was significant, Λ = .70, *F*(4, 48) = 5.10, *p*<.01, η_p_^2^ = .30. The MANOVA was followed up with discriminant analysis. This revealed one discriminant factor, canonical *R* ^2^ = .30, which significantly differentiated the cultural groups, χ^2^(4) = 17.35, *p*<.01. The correlations between outcomes and the discriminant function revealed that autonomous orientation (*r* = .81), personal focus (*r* = .32) and social interactions (*r* = −.78) loaded onto the function. Other-self ratio did not load onto this factor (*r* = .08). Hence, as in Study 1, the expected cultural differences were evident. Follow-up multiple univariate ANOVAs were also conducted. The results of these analyses are presented in Table 3.

### State Measures

A mixed 2 (group; East Asian vs. British) x 2 (time; pre-film vs. post-film) ANOVA found that participants scored significantly higher on the mood measures following the film, *F*(1, 51) = 50.33, *p*<.001. However, the interaction was also significant, *F*(1, 51) = 5.71, *p = *.02. As shown in Table 3, the East Asian and British groups did not differ in terms of pre-film mood but the East Asian group scored significantly higher on the post-film mood measures than the British group. The groups did not differ in terms of post-film distress and attention paid to the film (see Table 3).

### Intrusions of Film-Related Material

As in Study 1, the East Asian and British participants did not differ significantly regarding the number of film-related intrusions over the week following viewing the film as recorded in the diary (see Table 3). As in Study 1, the groups did not differ in terms of recognition and recall suggesting that objective memory performance was equally accurate across cultures (see Table 3). Given the groups were found to significantly differ in post-film mood scores, which may have had an influence on autobiographical remembering, the results were also conducted including post-film mood scores as a covariate. A similar pattern of results emerged.

As predicted, participants in Study 2 had significantly more intrusions (Study 1 *M = *4.10, *SD = *2.99; Study 2 *M = *8.83, *SD = *7.47), *t*(95) = 4.04, *p*<.001, *d = *0.85, and significantly worse recall (Study 1 *M = *10.53, *SD = *1.88; Study 2 *M = *9.26, *SD = *2.34), *t*(95) = 3.27, *p*<.01, *d = *0.68, and recognition scores (Study 1 *M = *10.75, *SD = *1.45; Study 2 *M = *9.69, *SD = *1.80), *t*(95) = 3.40, *p = *.001, *d = *0.70, than participants in Study 1. Participants in Studies 1 and 2 were not found to differ significantly in terms of any of the variables which may have influenced these findings (i.e. depression, remembering to complete diary, difference between pre- and post-film emotion, distress, attention paid to film and trauma exposure).

### Trauma Film Narratives

The means for trauma film memory-content variables are presented in Table 3. As shown in Table 3, the British group had significantly longer trauma film narratives than the East Asian group. A MANOVA with the memory-content variables (autonomous orientation, other-self ratio, social interactions) as the dependent variables was conducted. There was a significant effect of group on the memory-content variables, Λ = 0.83, *F*(3, 49) = 3.38, *p* = .03, η_p_^2^ = .17. A follow-up discriminant analysis revealed one discriminant factor, canonical *R* ^2^ = .17, which significantly differentiated the cultural groups, χ^2^ (3) = 9.30, *p = *.03. The correlations between outcomes and the discriminant function revealed that autonomous orientation (*r* = .73), social interactions (*r* = −.79) and other-self ratio (*r* = −.39) loaded onto the function. Follow-up multiple univariate ANOVAs were also conducted. The results of these analyses are presented in Table 3.

### Correlations between Trauma Film Narrative Properties and Film-Related Intrusions

Correlation analyses are shown in Table 4 (Bonferroni correction α = .017). For the British group a higher frequency of film-related intrusions correlated significantly with lower levels of autonomous orientation and self-focus in the trauma film narrative. For the East Asian group, a higher frequency of film-related intrusions correlated with higher levels of autonomous orientation in the trauma film narrative. The correlation coefficients differed significantly for autonomous orientation and other-self ratio.

**Table 4:** Summary of Correlation Coefficients between Trauma Film Memory-Content Variables and Number of Trauma Film-Related Intrusions (and Z score comparisons of the correlation coefficients) for each Group for Study 2.

	British Intrusions	East Asian Intrusions	*Z* score
Autonomous Orientation	−.73**	.39*	4.39**
Other-Self	.59**	−.07	2.49*
Social Interactions	.09	.11	0.10

*p<.05 **p<.01.

## Discussion

Study 2 replicated Study 1 and found that for the British group, a higher frequency of film-related intrusions was correlated significantly with lower levels of autonomous orientation and self-focus in the trauma film narrative. In contrast, for the East Asian group, a higher frequency of film-related intrusions correlated significantly with higher levels of autonomous orientation. These correlation coefficients differed significantly. Thus, Study 2 further supports the notion that integration and contextualization of memories (as indexed by evidence of culturally emphasized memory-content variables being present) of a trauma film is associated with fewer trauma film-related intrusions. Trauma memories that reflected culturally appropriate remembering were correlated significantly with fewer intrusions being experienced by an individual.

Second, as predicted, removal of the opportunity for participants to provide an immediate narrative of the trauma film post-viewing resulted in more intrusions and reduced performance on the recognition and cued-recall tasks one-week later. This suggests that developing a narrative immediately following viewing may allow for the film content to be rehearsed and may enhance conceptual post-memory integration. This finding is in line with Krans et al. [51] who found that by administering a verbal recognition memory test immediately post-viewing resulted in fewer trauma film-related intrusions and improved performance on the cued-recall memory test. Thus, direct efforts to allow participants to rehearse trauma-related material and thus, enhance conceptual post-memory integration may be associated with fewer trauma film-related intrusions.

Finally, unlike Study 1, we found cultural differences in memory-content variables of the delayed narrative. This demonstrated that cultural differences in self-construal acted as a reconstructive filter that shaped the memory over the course of retention and at the time of retrieval [34]. Therefore, it is possible that for tasks that are not specifically designed to encourage cultural effects, a delay period is required for cultural differences to emerge. Reactivation and rehearsal of the memory over the week may have strengthened the cultural influences on the memory [27]. Additionally, this period may have provided opportunities for the development of self-relevance and the generation of personal meaning of the material (which is likely to be more relevant for trauma material than a fictional story about a bear going to market). Participants may have associated the material with previous personal memories, daily activities and social events (e.g., conversations, news coverage, etc.) which may have encouraged cultural influences to be exerted on the memory. Further research is required to explore why Study 2 found cultural differences in the narrative but Study 1 did not and whether the removal of the initial narrative played a role in this difference.

## General Discussion

These two studies investigated the influence of culture on the relationship between the memory-content variables of the autobiographical remembering of trauma film material and film-related intrusions. Empirical work has demonstrated that the Western perspective of self-construal emphasizes autonomy, self-determination and self-expression in autobiographical remembering. In contrast, East Asian cultures discourage excessive self-focused, autonomously oriented remembering and rather focus on social interactions and others [33]. Verbal conceptual processing, integration and contextualization of the memory provides the required opportunities for cultural differences in self-construal to influence the way in which information is encoded and represented in memory. These processes also provide the required opportunities for cultural differences in self-construal to shape the memory over the course of retention and at the time of retrieval [34], [36]. Hence, evidence of culturally valued memory-content was taken as an index of integration and contextualization of the memory. In support of this, it was found that for British participants *lower* levels of expressions of self-determination and autonomy and self-focus (Studies 1 and 2) in the autobiographical remembering of the trauma film material was correlated significantly with a higher frequency of film-related intrusions. In contrast, for East Asian participants *higher* levels of self-determination and autonomy (Studies 1 and 2) and *less* focus on others (Study 1) were correlated significantly with a higher frequency of film-related intrusions. These findings suggest that those who contextualized and integrated the trauma film material in autobiographical memory may experience fewer intrusions than those who failed to contextualize and integrate the trauma film material in autobiographical memory. Thus, encouraging one to engage with the material in a way that aligns with one’s cultural self-construal and facilitating the process of narrative construction in a way that gives meaning and substance to one’s self-construal [34] may be effective in reducing trauma-related intrusive images.

Second, the findings suggest that developing a narrative task immediately post-film viewing may result in a reduction in film-related intrusions and improved performance on recognition and cued-recall tasks administered at one-week follow-up. This supports Krans et al.’s [51] findings. It suggests that performing a verbal conceptual type task immediately following viewing an aversive film may result in the film’s representations in memory being better contextualized and integrated with autobiographical memory. This may be because such a task reduces avoidance and enhances rehearsal of the material [51]. Kran et al. suggests that these findings are important because most of the empirical studies in this area focus on peri-traumatic processing of the trauma film material. Post-event information processing in intrusion development is important to consider given its theoretical relevance. Additionally, an important goal of PTSD treatments involves integrating the trauma representation in autobiographical memory. These findings further support Krans et al.’s suggestion that a simple intervention that facilitates engagement, rehearsal, conceptualization and integration of trauma material may be effective in reducing intrusive images. However, it is important to note that this research is still in its infancy and questions remain as to whether such a simple intervention would indeed be effective for highly traumatized individuals in a similar way to that demonstrated in these studies.

Study 2 also demonstrated cultural differences in the memory-content qualities of the trauma film. Research has demonstrated that cultural differences in autobiographical remembering reflect more overarching differences in general narrative (i.e. extends to fictional stories) reporting rather than being unique to personal narratives [27], [34]. The current research has extended this to the trauma film paradigm. It was found that while objective story memory performance of the trauma film material was equally accurate across cultures, memory-content of the accounts provided one-week following viewing culturally differed. The British group emphasized autonomous orientation and self-focus. The East Asian group had greater emphasis on social interactions than the British group. This supports the notion that cultural differences in self-construal act as a reconstructive filter [34]. It is worth noting, however, that cultural differences were not evident in the immediate or delayed narratives in Study 1. It is uncertain why this was the case. It is possible that when employing a task that was not explicitly designed to elicit cultural effects, a delay may be required to allow personal interpretations of events to emerge and cultural influences to be exerted on the memory. In support of this, in Study 1 the correlations between the memory-content variables and frequency of intrusions were only found for the delayed narrative and not the immediate narrative. The question emerges however, why cultural differences were not then evident in the delayed narrative in Study 1. It is possible that the immediate narrative in some way disrupted the required processes. Future research is required to explore this further.

The present studies took a novel approach to examining the influence of culture on the development and maintenance of PTSD. Specifically, this is the first study to use an analogue approach to investigate the influence of culture on the development of intrusive memories. There is increasing recognition that PTSD is observed in many different societies and cultures. However, there is currently very limited understanding of the processes involved in PTSD in those from non-Western cultural backgrounds [52]. This is an important area to consider empirically. Cross-cultural research has demonstrated that cultural differences in self-construal influence the very processes (e.g., information-processing and autobiographical remembering) posited by PTSD models to be involved in PTSD (see [36]). Thus, the question arises; do cultural differences in information-processing and autobiographical remembering influence the development of intrusive PTSD memories? The current findings suggest that while intrusive memories may develop in a universally similar way, culture may influence the way trauma memories are integrated and contextualized. For instance, while a deeper, personal, meaningful processing of the material may protect against intrusions, what is deemed personal and meaningful may culturally differ.

It is important to highlight that the cultural interpretations derived from the findings of two studies were based on correlational analyses. Therefore, while the hypotheses were theoretically-derived, causation cannot be inferred. It is possible that other factors may have influenced and accounted for the results. For instance, scores on memory variables that are counter to the prevailing cultural norm may indicate the presence of distress or psychopathology in an individual. For example, British participants who score low on autonomy may be more anxious in general. This in turn may have resulted in a higher number of trauma film-related intrusions being reported by these individuals. In response to this possibility, for both studies correlation analyses were conducted to examine the associations between memory-content variables in the personal narratives and subsequent intrusions of the trauma film. In each instance the correlation analyses were found to be non-significant. Thus there was no evidence to suggest that levels of memory-content variables in general personal remembering was associated with the number of intrusions reported by participants. Nevertheless, it is important that future research further explore the assertions outlined in these studies.

There are several additional shortcomings associated with the current research. First, the choice for using the trauma film paradigm as an analogue of real-life trauma is appropriate in the current studies. However, there are significant limitations associated with this paradigm in regards to whether the findings extend to real life trauma experiences. Second, the size and nature (university sample) of the sample potentially limits the generalizability and clinical interferences of such findings. Third, as in any study exploring the influence of culture on certain variables, language, task understanding and length of time in host culture must be considered. The finding of no cultural differences in task difficulty and participants having university-standard English language were taken collectively to suggest there were no major differences in task understanding and responding. Additionally, the mean length of time East Asian participants had lived in the UK was less than two years. A similar pattern of results also emerged when language ability and length of time in the UK were included as covariates in the analyses. Furthermore, research has suggested that East Asian and Western identities may be stored in separate knowledge structures in bicultural individuals, with each structure activated by its associated language [53]. Thus, future research would benefit from ensuring participants completed the memories in their first language. However, East Asian participants providing the memories in their first language should result in more pronounced differences. Fourth, the Western cultural environment that this study was conducted in and the international student status of the East Asian group may have influenced findings. Fifth, the East Asian group was comprised of participants from several East Asian cultures. While such homogeneous groups have been used in previous cultural autobiographical memory research (e.g. [34]), it is worth exploring East Asian cultures separately in future research. Finally, it is possible that cultural differences were the result of cultural differences in the norms of what should be reported when recalling a story rather than differences in memory representations [31]. However, other research has demonstrated that cultural differences in memory reports do reflect differences in the ways people remember autobiographical events rather than merely the ways they write or talk about these events (e.g. [34]). Despite these limitations, this is the first study to employ an analogue paradigm to investigate the influence of culture on the development of intrusive trauma memories. The findings suggest that greater integration and contextualization of the trauma memory is associated with fewer intrusions and the more the trauma memory reflects culturally appropriate remembering, the fewer intrusions.

## References

[1] Johannessen KB, Berntsen D (2010) Current concerns in involuntary and voluntary autobiographical memories. Consciousness & Cognition 19: 847–860. 20188597 10.1016/j.concog.2010.01.009

[2] Berntsen D (1996) Involuntary autobiographical memories. Applied Cognitive Psychology 10: 435–454.

[3] Deeprose C, Zhang S, Dejong H, Dalgleish T, Holmes EA (2012) Imagery in the aftermath of viewing a traumatic film: using cognitive tasks to modulate the development of involuntary memory. Journal of Behaviour Therapy Experimental Psychiatry 43: 758–764. 10.1016/j.jbtep.2011.10.008 PMC3545201 22104657

[4] Holmes EA, Bourne C (2008) Inducing and modulating intrusive emotional memories: A review of the trauma film paradigm. Acta Psychologica 127: 553–566. 18234153 10.1016/j.actpsy.2007.11.002

[5] Rubin DC, Berntsen D (2009) The frequency of voluntary and involuntary autobiographical memories across the life span. Memory Cognition 37: 679–688. 19487759 10.3758/37.5.679 PMC3044938

[6] Brewin CR (2014) Episodic memory, perceptual memory, and their interaction: Foundations for a theory of posttraumatic stress disorder. Psychological Bulletin 140: 69–97. 23914721 10.1037/a0033722

[7] Brewin CR, Dalgleish T, Joseph S (1996) A dual-representation theory of posttraumatic stress disorder. Psychological Review 106: 670–686. 10.1037/0033-295x.103.4.670 8888651

[8] Ehlers A, Clark DM (2000) A cognitive model of posttraumatic stress disorder. Behaviour Research & Therapy 38: 319–345. 10761279 10.1016/s0005-7967(99)00123-0

[9] Conway MA (2005) Memory and the self. Journal of Memory and Language 53: 594–628.

[10] Conway MA, Pleydell-Pearce CW (2000) The construction of autobiographical memories in the self-memory system. Psychological Review 107: 261–288. 10789197 10.1037/0033-295x.107.2.261

[11] Dalgleish T (2004) Cognitive theories of posttraumatic stress disorder: The evolution of multi-representational theorizing. Psychological Bulletin 130: 228–260. 14979771 10.1037/0033-2909.130.2.228

[12] Brewin CR, Gregory JD, Lipton M, Burgess N (2010) Intrusive images in psychological disorders: Characteristics, neural mechanisms, and treatment implications. Psychological Review 117: 210–232. 20063969 10.1037/a0018113 PMC2834572

[13] American Psychiatric Association (2004) Diagnostic and statistical manual of mental Disorders-IV-TR. Washington DC: Author.

[14] Bourne C, Frasquilho F, Roth AD, Holmes EA (2010) Is it mere distraction? Peri-traumatic verbal tasks can increase analogue flashbacks but reduce voluntary memory performance. Journal of Behaviour Therapy Experimental Psychiatry 41: 316–324. 10.1016/j.jbtep.2010.03.001 20359691

[15] Brewin CR, Saunders J (2001) The effect of dissociation at encoding on intrusive memories for a stressful film. British Journal of Medical Psychology 74: 467–472. 11780794 10.1348/000711201161118

[16] Holmes EA, Brewin CR, Hennessy RG (2004) Trauma Films, Information Processing, and Intrusive Memory Development,10.1037/0096–3445.133.1.3, Journal of Experimental Psychology-general. 133: 3–22. 10.1037/0096-3445.133.1.3 14979748

[17] Krans J, Näring G, Holmes EA, Becker ES (2010) Motion effects on intrusion development. Journal of Trauma and Dissociation 11: 73–82. 20063249 10.1080/15299730903318483

[18] Krans J, Näring G, Holmes EA, Becker ES (2010) “I see what you’re saying”: Intrusive images from listening to a traumatic verbal report. Journal of Anxiety Disorders 24: 134–140. 19864108 10.1016/j.janxdis.2009.09.009

[19] Stuart ADP, Holmes E A, Brewin CR (2006) The influence of a visuospatial grounding task on intrusive images of a traumatic film. Behavior Research and Therapy 44: 611–619. 10.1016/j.brat.2005.04.004 15979563

[20] Holmes E, James EL, Kilford EJ, Deeprose C (2010) Key steps in developing a cognitive vaccine against traumatic flashbacks: Visuospatial Tetris versus verbal Pub Quiz. PLoS ONE 5, e13706. doi: 10.1371/journal.pone.0013706. 10.1371/journal.pone.0013706 PMC2978094 21085661

[21] Nixon RDV, Cain N, Nehmy T, Seymour M (2009) The influence of thought suppression and cognitive load on intrusions and memory processes following an analogue stressor. Behavior Therapy 40: 368–379. 19892082 10.1016/j.beth.2008.10.004

[22] Nixon RDV, Nehmy T, Seymour M (2007) The effect of cognitive load and hyperarousal on negative intrusive memories. Behaviour Research and Therapy 45: 2652–2663. 17666185 10.1016/j.brat.2007.06.010

[23] Krans J, Näring G, Becker ES (2009) Count out your intrusions: Effects of verbal encoding on intrusive memories. Memory 17: 809–815. 19657961 10.1080/09658210903130780

[24] Logan S, O’Kearney R (2012) Individual differences in emotionality and peri-traumatic processing. Journal of Behavior Therapy and Experimental Psychiatry 43: 815–822. 22197753 10.1016/j.jbtep.2011.12.003

[25] Markus HR, Kitayama S (2010) Cultures and selves: A cycle of mutual constitution. Perspectives on Psychological Science 5: 420–430. 26162188 10.1177/1745691610375557

[26] Lee LC, Zane NWS (1998) Handbook of Asian American Psychology. Thousand Oaks, CA: Sage.

[27] Han JJ, Leichtman MD, Wang Q (1998) Autobiographical memory in Korean, Chinese, and American children. Developmental Psychology 34: 701–713. 9681262 10.1037//0012-1649.34.4.701

[28] Park DC, Huang C-M (2010) Culture wires the brain: A cognitive neuroscience perspective. Perspectives on Psychological Science 5: 391–400. 22866061 10.1177/1745691610374591 PMC3409833

[29] Jobson L, O’Kearney RT (2008) Cultural differences in retrieval of self-defining memories. Journal of Cross-Cultural Psychology 39: 75–80.

[30] Wang Q (2008) Emotion knowledge and autobiographical memory across the preschool years: A cross-cultural longitudinal investigation. Cognition 108: 117–135. 18353299 10.1016/j.cognition.2008.02.002

[31] Wang Q, Conway MA (2004) The stories we keep: Autobiographical memory in American and Chinese middle-aged adults. Journal of Personality 72: 911–938. 15335332 10.1111/j.0022-3506.2004.00285.x

[32] Wang Q, Leichtman MD, Davies KI (2000) Sharing memories and telling stories: American and Chinese mothers and their 3-year-olds. Memory 8: 159–178. 10889900 10.1080/096582100387588

[33] Ross M, Wang Q (2010) Why we remember and what we remember: Culture and autobiographical memory. Perspectives on Psychological Science 5: 401–409. 26162186 10.1177/1745691610375555

[34] Wang Q, Ross M (2005) What we remember and what we tell: The effects of culture and self-priming on memory representations and narratives. Memory 13: 594–206. 16076674 10.1080/09658210444000223

[35] Conway MA, Jobson L (2012) On the nature of autobiographical memory. In Berntsen D, Rubin DC, editors. Understanding autobiographical memory: Theories and approaches. Cambridge: Cambridge University Press.

[36] Jobson L (2009) Drawing current posttraumatic stress disorder models into the cultural sphere: The development of the conceptual self model. Clinical Psychology Review 29: 368–381. 19344988 10.1016/j.cpr.2009.03.002

[37] Holmes EA, James EL, Coode-Bate T, Deeprose C (2009) Can Playing the Computer Game ‘Tetris’ Reduce the Build-up of Flashbacks for Trauma?’ A Proposal from Cognitive Science. PLoS ONE, 4(1), e4153. doi: 10.1371/journal.pone.0004153. 10.1371/journal.pone.0004153 PMC2607539 19127289

[38] Foa EB, Riggs DS, Dancu CV, Rothbaum BO (1993) Reliability and validity of a brief instrument for assessing posttraumatic stress disorder. Journal of Traumatic Stress 6: 459–473.

[39] Derogatis LR, Lipman RS, Rickels K, Cori L (1974) The Hopkins Symptom Checklist (HSCL): A self-report symptom inventory. Behavioral Science 19: 1–15. 4808738 10.1002/bs.3830190102

[40] Mollica RF, Wyshak G, de Marneffe D, Khuon F, Lavelle J (1987) Indochinese versions of the Hopkins Symptom Checklist-25: A screening instrument for the psychiatric care of refugees. American Journal of Psychiatry 144: 497–500. 3565621 10.1176/ajp.144.4.497

[41] Hagenaars MA, Van Minnen A, Holmes EA, Brewin CR, Hoogduin CAL (2008) The effect of hypnotically-induced somatoform dissociation on intrusion development after an aversive film: An experimental study. Cognition and Emotion 22: 944–963.

[42] Schartau PE, Dalgleish T, Dunn BD (2009) Seeing the bigger picture: Training in perspective broadening reduces self-reported affect and psychophysiological response to distressing films and autobiographical memories. Journal of Abnormal Psychology 118: 15–27. 19222310 10.1037/a0012906

[43] Jobson L (2011) Cultural differences in levels of autonomous orientation in autobiographical remembering in posttraumatic stress disorder. Applied Cognitive Psychology 25: 175–182.

[44] Kuhn MH, McPartland TS (1954) An empirical investigation of self-attitudes. American Sociological Review 19: 68–76.

[45] Wang Q (2001) Culture effects on adults’ earliest childhood recollection and self- description: Implications for the relation between memory and the self. Journal of Personality and Social Psychology 81: 220–233. 11519928 10.1037//0022-3514.81.2.220

[46] Trafimow D, Triandis H, Goto S (1991) Some tests of the distinction between the private and collective self. Journal of Personality and Social Psychology 60: 649–655.

[47] Triandis H (1989) The self and social behavior in differing social contexts. Psychological Review 96: 506–520.

[48] Wang Q, Leichtman MD (2000) Same beginnings, different stories: A comparison of American and Chinese children’s narratives. Child Development 71: 1329–1346. 11108099 10.1111/1467-8624.00231

[49] Wang Q, Leichtman MD, White SH (1998) Childhood memory and self-description: The impact of growing up an only child. Cognition 69: 73–103. 9871372 10.1016/s0010-0277(98)00061-4

[50] Field A (2009) Discovering statistics using SPSS: Introducing Statistical Methods. London UK: SAGE.

[51] Krans J, Näring G, Holmes EA, Becker ES (2009) Tell me more: Can a memory test reduce analogue traumatic intrusions? Behaviour Research and Therapy 47: 426–430. 19232572 10.1016/j.brat.2009.01.009

[52] Foa EB, Keane TM, Friedman M J, Cohen JA (2009). Effective treatments for PTSD: Practice guidelines from the International Society for Traumatic Stress Studies (2nd ed.). New York: Guilford Press.

[53] Ross M, Xun WQE, Wilson AE (2002) Language and the bicultural self. Personality and Social Psychology Bulletin 28: 1040–1050.

